# Beyond dry eye: The greater extent of Sjögren's systemic disease symptoms, the impact of COVID‐19 and perceptions towards telemedicine identified through a patient co‐designed study

**DOI:** 10.1111/hex.13823

**Published:** 2023-07-20

**Authors:** Emily Greenan, Gráinne Tynan, Deirdre Collins, Conor C. Murphy, Michelle Flood, Joan Ní Gabhann‐Dromgoole

**Affiliations:** ^1^ School of Pharmacy and Biomolecular Sciences RCSI, University of Medicine and Health Sciences Dublin Ireland; ^2^ Department of Ophthalmology RCSI University of Medicine and Health Sciences Dublin Ireland; ^3^ Royal Victoria Eye and Ear Hospital Dublin Ireland; ^4^ Sjögren's Ireland Dublin Ireland; ^5^ RCSI PPI Ignite Network RCSI, University of Medicine and Health Sciences Dublin Ireland

**Keywords:** diagnosis, disease, public patient involvment, Sjögren's, telemedicine

## Abstract

**Background:**

Sjögren's (‘SHOW‐grins’) is a chronic debilitating autoimmune disease characterised by dry eyes and dry mouth, secondary to reduced exocrine function of both the lacrimal and salivary glands. The persistent, severe and serious systemic complications of Sjögren's are poorly understood and often unappreciated, resulting in significant morbidity and treatment burden. This study aimed to explore the experiences of those living with Sjögren's, specifically access to healthcare and attitude towards telemedicine. Additionally, we sought to collect information regarding the impact of the pandemic on their quality of life (QoL).

**Methods:**

One hundred and ninety‐four individuals attended an Irish Sjögren's Webinar. Attendees were invited to participate in two online surveys after the webinar. The first survey gathered information related to demographics, disease and experiences during the COVID‐19 pandemic. A combination of bespoke items and validated questionnaires (EULAR Sjögren's Syndrome Patient Reported Index [ESSPRI], COVID‐19 Impact on Quality of Life [COV19‐QoL]) was used. The second survey consisted of a shortened Telehealth Usability Questionnaire. Both were prepared in collaboration with a patient advocate.

**Results:**

Survey 1: *n* = 76; response rate = 39.2%. Thirty‐one respondents (41.4%) to survey 1 reported a delay of ≥5 years between the onset of symptoms and diagnosis. Dry mouth was the most common symptom experienced (76.8%, *n* = 63), followed by dry eye (74.4%, *n* = 61), fatigue (57.3%, *n* = 47) and joint pain (53.7%, *n* = 44), but a range of other symptoms were also reported. COV19‐QoL results indicated that the pandemic had a detrimental effect on participants' overall QoL (4.0 ± 1.0) and physical health (4.0 ± 0.8) in particular. COV19‐QoL and ESSPRI scores were moderately correlated (0.36, *p* = .002). Over 70% of respondents had a medical appointment cancelled, delayed or rescheduled (*n* = 60). Survey 2: *n* = 57; response rate = 29.4%. Those that had interacted with telemedicine reported largely positive experiences with the virtual model.

**Conclusion:**

Clinicians should be aware of the range of symptoms experienced by patients with Sjögren's beyond those of sicca (dry eye and dry mouth) and fatigue. COVID‐19 has negatively influenced the self‐reported health and well‐being of those with Sjögren's, particularly those with higher symptom scores. It is vital that optimised telemedicine models are implemented to ensure continuity in the provision of healthcare for those with chronic illness such as Sjögren's and in preparation for possible future pandemics.

**Patient or Public Contribution:**

A group of people living with Sjögren's co‐designed the structure and content of the webinar where the survey was shared. A public and patient involvement (PPI) contributor also collaborated in the selection of questionnaires used in the study, ensuring that the questions asked would best reflect the priorities of patients. They contributed to the writing of this manuscript as co‐authors. Additionally, the research team and Sjögren's patients who contributed to this work have gone on to establish Sjögren's Research Ireland, a collaboration between patient advocates, researchers and PPI facilitators.

## 1 INTRODUCTION

Sjögren's (‘SHOW‐grins’) is a debilitating autoimmune disease that affects approximately 1 in 200 people.^1^, ^2^ Traditionally, Sjögren's has been considered a localised disease of the exocrine glands, where chronic inflammation, accompanied by increased lymphocytic infiltration of the lacrimal and salivary glands, results in severely dry eyes and dry mouth, often referred to as ‘sicca’ or sicca symptoms.^3^ However, in addition to these pathological processes, which lead to exocrine gland dysfunction and ultimately gland destruction,^4^, ^5^ those living with Sjögren's can also develop a wide range of systemic clinical manifestations that can affect essentially any organ system. Systemic extra glandular complications, typically recognised 5–10 years after initial diagnosis, occur in 20%–40% of patients and can include profound fatigue,^6^ chronic joint and muscle pain,^7^, ^8^ skin rashes, increased incidence of lymphoma^9^, ^10^ and pulmonary complications which range from mild reduction in lung function to severe complications such as idiopathic pulmonary fibrosis.^11^ Sjögren's is most commonly diagnosed between the ages of 40 and 60, with women nine times as likely to be diagnosed with Sjögren's than men.^1^, ^2^

Recently, there has been an international trend, stemming from patient–researcher–clinician partnerships, towards moving away from the term ‘Sjögren's Syndrome’ to ‘Sjögren's’, ‘Sjögren's Disease’ or Sjögren's (in association with).^12^, ^13^ It is expected that this shift in nomenclature would support the definition of Sjögren's as a serious disease having a known cause and distinct symptoms, rather than a syndrome that has connotations of nuisance symptoms rather than a distinct autoimmune disease with serious outcomes.^12^ Furthermore Kollert et al. suggest abandoning the terms ‘primary’ and ‘secondary’ Sjögren's (previously used to indicate whether Sjögren's is the only diagnosis or if it is a complication of another autoimmune disease) in an attempt to consider Sjögren's holistically without the implied hierarchy of primary versus secondary, which often diminishes the importance of Sjögren's when co‐occurring with another autoimmune condition.^12^, ^13^, ^14^

Critical appraisal of the field over the last few decades finds that treatment approaches for Sjögren's remain reactionary rather than curative; therapies have evolved to treat symptoms rather than the underlying pathology.^15^, ^16^ Significant delays from initial symptoms to diagnosis and lack of effective treatments result in irreversible fibrosis to the exocrine glands.^17^ While previous studies have suggested that Sjögren's is a growing public health concern that significantly impacts the quality of life (QoL) (including physical, social, psychological functioning, daily activities and workplace productivity),^18^, ^19^, ^20^ the paucity of research focusing on the ocular surface and systemic components of this disease has perpetuated the lack of appreciation regarding the significant impact of Sjögren's on patients QoL amongst scientists and clinicians.^21^

Furthermore, people living with Sjögren's were considered particularly at risk from severe acute respiratory syndrome coronavirus 2 (COVID‐19), the outbreak of which led to the declaration of a global pandemic by the World Health Organization in March 2020.^22^ The majority of cases are characterised by mild symptoms; however, those with autoimmune disease are considered to be at risk for more severe disease. Due to the underlying immune dysfunction and frequent use of immunosuppressive medications, patients with autoimmune conditions like Sjögren's are more likely to suffer from poor outcomes subsequent to COVID‐19 infection.^23^, ^24^ On 29 February 2020, the first case of the COVID‐19 virus was reported in Ireland.^25^ As of June 2023, there have been over 1,713,340 cases reported and over 9028 deaths in Ireland.^26^ The Irish public health response has involved containment through the implementation of a combination of counters measures including ‘lockdowns’, a national vaccination programme, and a reduction of nonessential healthcare services to ensure the provision of acute and COVID‐19‐related care. Furthermore, in an effort to reduce transmission, telemedicine appointments often took the place of face‐to‐face consultations, providing remote patient assessment and provision of care.

Therefore, the research team was interested in exploring (1) the patient‐reported experience of living with Sjögren's, (2) the reported impact of the COVID‐19 pandemic on self‐perceived QoL and health service use and (3) attitudes towards telemedicine.

## 2 METHODS

The Research and Ethics Committees of RCSI, University of Medicine and Health Sciences granted ethical approval for this survey study (REC 202105006). The study adhered to the tenets of the Declaration of Helsinki. Informed consent was obtained from all participants before participation.

### 2.1 Participants and study design

**Figure 1:**
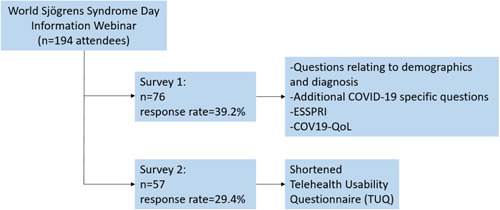
Schematic outlining the components of each of the surveys sent to attendees of the World Sjögren's Day Information Webinar. COV19‐QoL, COVID‐19 Impact on Quality of Life; ESSPRI, EULAR Sjögren's Syndrome Patient Reported Index.

A nonprobability, purposive sampling method was used that aimed to identify participants with relevant experience. All attendees (*n* = 194) of a virtual Irish Sjögren's Syndrome Information Webinar held on World Sjögren's Day on 23 July 2021 were invited to complete two online questionnaires after the webinar via Survey Monkey. In each instance, attendees were sent the SurveyMonkey® link, followed by weekly reminders for 4 weeks before the surveys were closed and the data were collected (Figure 1).

As part of the first survey, questions were included about (1) demographics, (2) Sjögren's symptoms (via the validated EULAR Sjögren's Syndrome Patient Reported Index [ESSPRI] and free text questions about the most impactful disease symptoms), priorities for future research and (3) the impact of the global pandemic via the COVID‐19 Impact on Quality of Life scale (COV19‐QoL) validated questionnaire and additional questions about their experiences of healthcare. As part of our second survey 2, we explored participants perspectives on telehealth using a modified (shortened) version of the Telehealth Usability Questionnaire (TUQ) validated questionnaire. Questionnaire design decisions are described in more detail in  Section 2.3.

### 2.2 Public and patient involvement (PPI) in study design

Before dissemination, surveys were critically reviewed in collaboration with a PPI contributor. In each instance, the patient advocate was asked to appraise the surveys in relation to accessibility and inclusion, relevance, necessity, language and overall questionnaire length. Adjustments were made in response to feedback and included recommendations to shorten the surveys to reduce the amount of screen time required, due to common Sjögren's symptoms of dry eye, fatigue and brain fog.

### 2.3 Questionnaires

#### 2.3.1 Demographic questions

Participants were asked to indicate their gender and age.

#### 2.3.2 Sjögren's diagnosis and current symptoms (ESSPRI and open question)

Participants were asked to indicate their time since diagnosis and time to diagnosis in years. The next section included the ESSPRI questionnaire, which is designed to measure the three main symptoms of primary Sjögren's syndrome (pSS): dryness, fatigue and pain. They are rated on global assessment scales from 1 to 10, with a higher score indicating more severe symptoms.^27^ This was chosen as it is a commonly used validated tool and supported the achievement of the study goals.

Participants were also asked an open question about what disease symptoms impacted their day‐to‐day lives the most. Responses were collated and presented in table format and as a Word Cloud using Microsoft Excel to collate the data and RStudio 4.3.1 to generate the image. Through discussion between patient advocates and researchers via Zoom, responses with different wording for similar symptoms were combined, for example, with dry eye, sicca, severe dry eye, dry eyes and extreme dry eye were included as dry eye. Each response was assigned a value of 1. The overall score for a particular symptom was determined by summing its frequency amongst participant responses, followed by the calculation of this symptom as a percentage of the number of respondents. Sizing is based on the frequency of each symptom, the larger the word appears, the greater the number of survey participants who indicated this was a symptom that they experienced daily. While a word cloud is not a common way to present such data, this format was believed to be more accessible by patient advocates.

Finally, respondents were asked their perspectives on what would be required to improve their care. A list of five options (Diagnosis, Effective therapies, Mental health, Patient education, Physician education) was agreed through discussion amongst the research team informed by the wider literature. Participants could select one or more of the available options.

#### 2.3.3 Impact of COVID‐19 on QoL and patient experience

To explore experiences during the pandemic more specifically, the recently developed COV19‐QoL questionnaire was included. It assesses respondents' QoL in the preceding week during the COVID‐19 pandemic.^28^ It had been suggested in the commentary about COVID‐19 that those with autoimmune conditions were experiencing reduced QoL and mental health as a result of the pandemic and we wanted to include this in the study. The questionnaire has six items, rated on a 5‐point Likert scale and the score is calculated by dividing the total score by the number of items. A higher score indicates that the perceived effect of the pandemic on a person's QoL is greater.

Additional questions were asked to further understand the personal experiences of participants during the COVID‐19 pandemic. These are outlined in Table 1 below. They included questions based on experiences in other jurisdictions, including experience and impact of medication shortages and interruptions, and the impact of mask wearing on sicca symptoms.^27^, ^28^, ^29^

**Table 1:** Outlining additional questions posed to participants to measure the personal impact of the COVID‐19 pandemic on participants' daily life.

Question	Answer options
Did you have medical appointments affected during the COVID‐19 pandemic?	Yes/No
Please indicate if these were cancelled/delayed/rescheduled	Multiple choice
Please indicate what specialties the appointments related to	Open answer
Over the past year did you experience disruptions to your prescribed medication regime?	Yes/No
If you take immunosuppression medication, did you reduce or discontinue this over the past year?	Yes/No
Was COVID a factor in this decision?	Yes/No
Have you noticed a worsening of sicca symptoms due to wearing a mask?	Yes/No

#### 2.3.4 TUQ

One of the most significant changes to healthcare delivery during the pandemic was the transition to online or ‘telehealth’ delivery for the majority of consultations to reduce the risk of transmission in healthcare facilities. Therefore, the team identified the importance of capturing this as part of the project via a second survey. The most commonly used telemedicine questionnaires include the TUQ (19%),^29^ Telemedicine Satisfaction Questionnaire (13%)^30^ and Service User Technology Acceptability Questionnaire (5.5%)^31^ according to a recent review.^32^ The TUQ was deemed most suitable as it broadly evaluates the usability of telehealth services, it has 21 items based on 6 criteria including usefulness (3 items), ease of use and learnability (3 items), interface quality (4 items), interaction quality (4 items), reliability (3 items) and satisfaction and future use (4 items).^29^

The validated TUQ traditionally consists of 21 items.^29^, ^33^ However, due to visual symptoms and reduced screen capacity time experienced by many people living with Sjögren's, the research team prepared a shortened version that was administered as part of the second online survey that focused on Telehealth.

### 2.4 Statistical analysis

Statistical analysis of data was performed using GraphPad Prism 9.0 for Windows (GraphPad Software). Data are expressed as the mean ± standard deviation, and ranges are presented where indicated. The normality of each variable was tested, and nonparametric analyses were computed using Spearman *r* formulations and Mann–Whitney tests.

## 3 RESULTS

### 3.1 Demographics

Seventy‐six attendees of the webinar (39.2%) responded to the initial online survey. Respondents were predominantly female (female 97%, *n* = 42; male 3%, *n* = 2), with a mean age of 51.6 years (range 19−73 years). The majority of respondents (53.2%, *n* = 42) had a diagnosis of pSS and a smaller number had a diagnosis of secondary Sjögren's syndrome (8.9%, *n* = 7), and others were unsure (32.9%, *n* = 27).

### 3.2 Sjögren's diagnosis and symptoms

The majority of respondents had their Sjögren's diagnosis for 1–5 years (51.3%, *n* = 39) and it was most common to have experienced symptoms for 1–5 years before receiving their diagnosis from a medical professional (38.7%, *n* = 29). Just over a quarter (26.7%, *n* = 20) of survey respondents reported a delay of >10 years between symptom onset and diagnosis.

**Figure 2:**
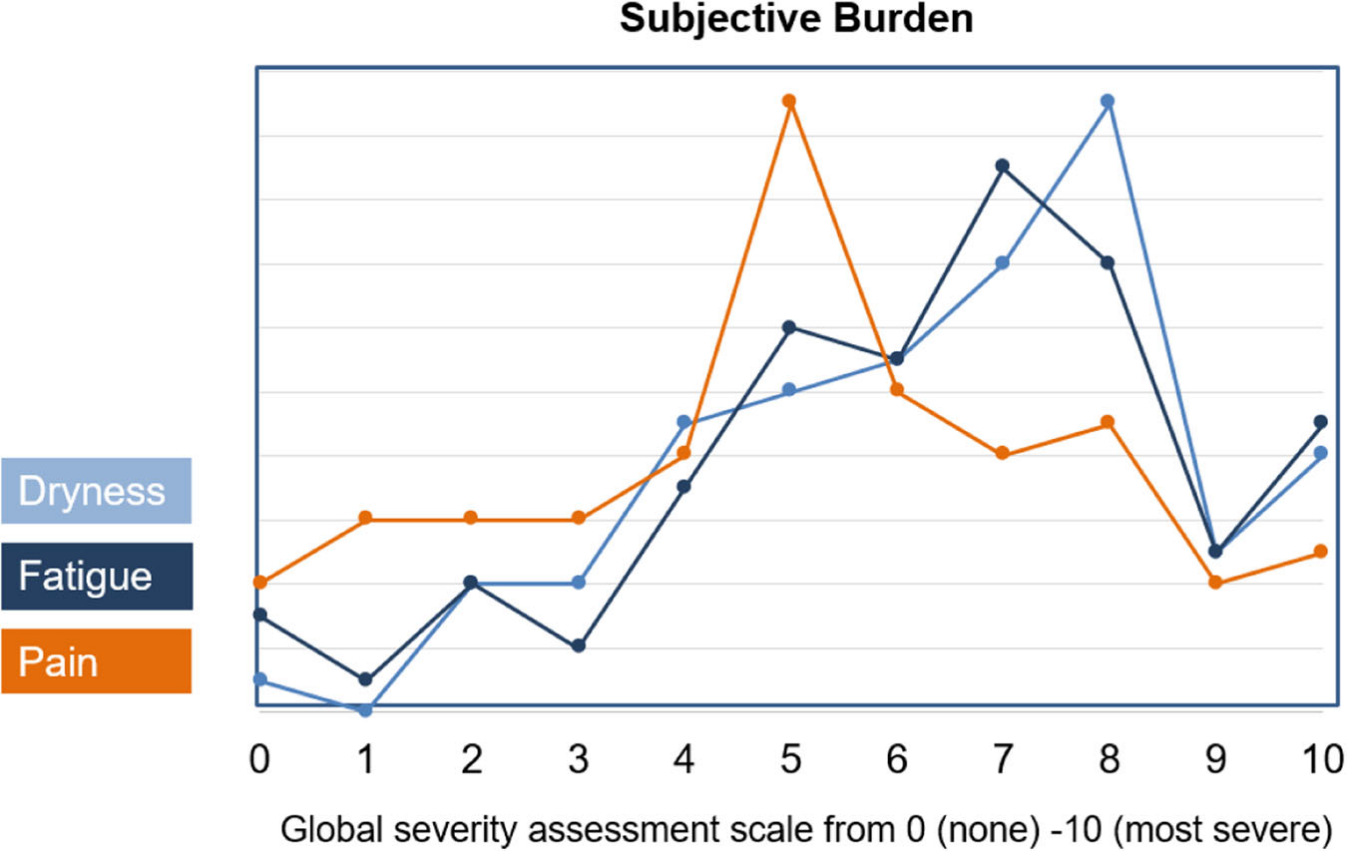
Summary of EULAR Sjögren's Syndrome Patient Reported Index.

In relation to participant responses to the ESSPRI questionnaire, the mean dryness, fatigue and pain scores were 6.6 ± 2.0, 6.6 ± 2.2 and 5.3 ± 2.2, respectively, and the mean total score was 18.4 ± 2.0 (Figure 2).

**Figure 3:**
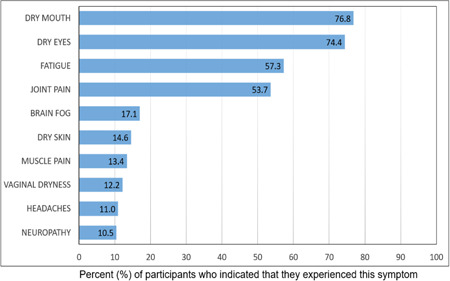
Chart representing the most prevalent symptoms experienced by survey respondents. Data are represented as a percentage of survey respondents who indicated that they experienced the symptom indicated.

Participants were also asked what disease symptoms they found the most troublesome day to day, the results of which were summarised in a bar chart (Figure 3). Overall, respondents attributed 100 symptoms to their Sjögren's. Dry mouth was the most common symptom experienced by respondents (76.8%, *n* = 63), followed by dry eyes (74.4%, *n* = 61), fatigue (57.3%, *n* = 47) and joint pain (53.7%, *n* = 44).

**Figure 4:**
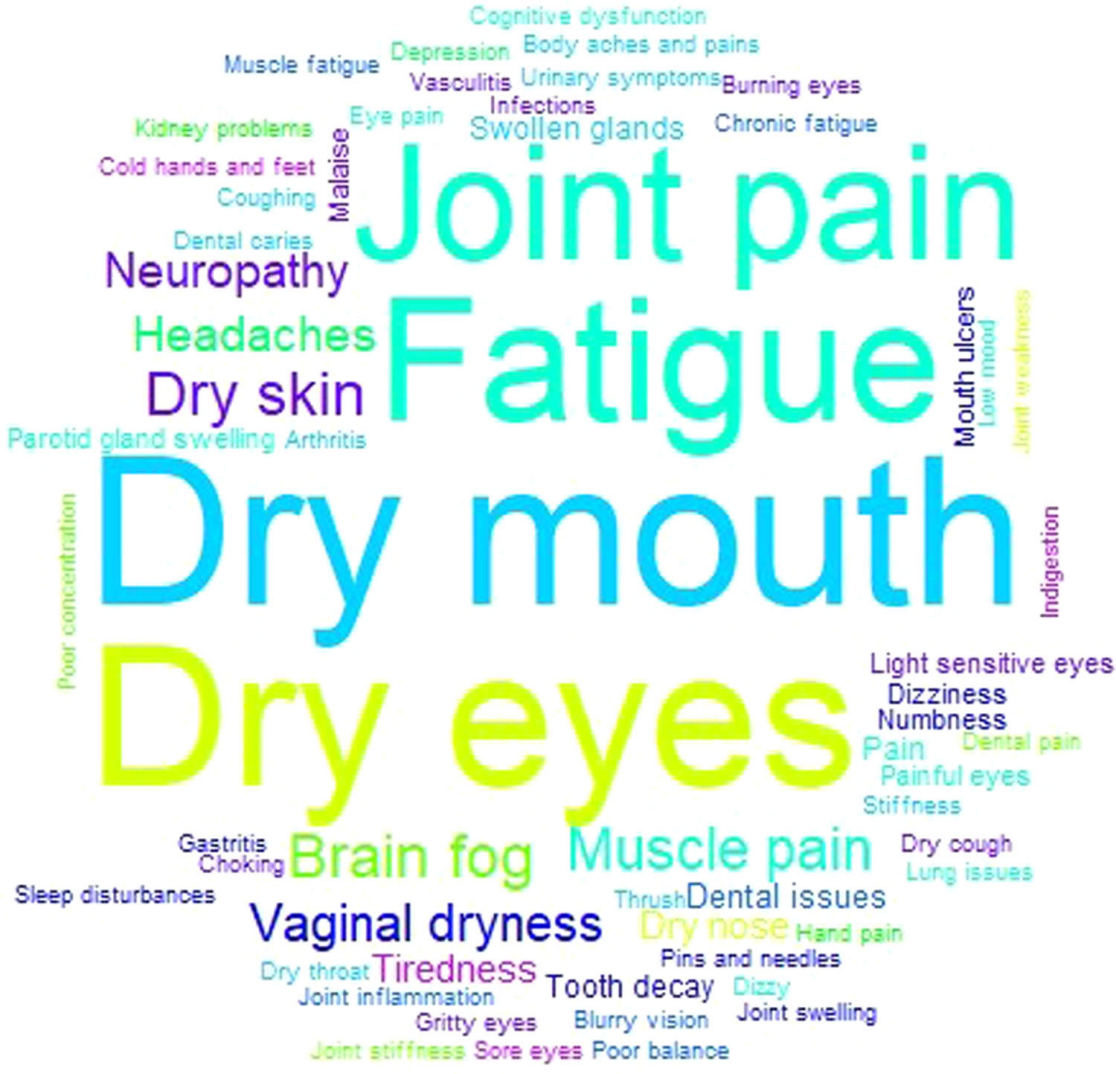
Common symptoms experienced by individuals with Sjögren's.

The spectrum of symptoms reported by respondents is shown in Figure 4 as a word cloud with the size of the words representing their relative frequency in responses.

Word cloud illustrating the most dominant symptoms of disease experienced by participants. The larger the word appears, the greater the number of survey participants who indicated this was a symptom that they experienced daily.

**Figure 5:**
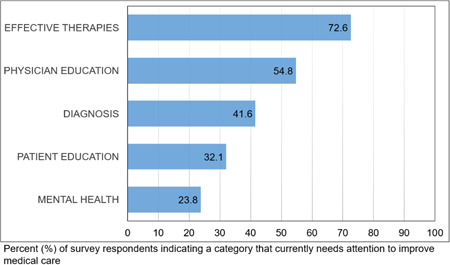
Areas that currently need attention to improve medical care. Data are represented as percent of survey respondents selecting an option from the choices indicated.

In terms of priorities for improving patient care, 72.6% (*n* = 61) of survey respondents indicated effective therapies as one of the major areas that scientists and healthcare providers need to focus on to improve the medical care of Sjögren's patients. Over half of the survey respondents (54.8%, *n* = 46) indicated a requirement to improve education amongst healthcare professionals as one of their top priorities. The diagnosis was selected by 41.6% of respondents, patient education was selected by 32.1% (*n* = 27) of respondents and mental health was selected by 23.8% (*n* = 20) of respondents (Figure 5).

### 3.3 Impact of COVID‐19

In the COV19‐QoL questionnaire, participants scored the most highly (indicating higher levels of concern) in relation to their perception of their overall QoL (4.0 ± 1.0), physical health (4.0 ± 0.8) and feeling more tense than before (3.7 ± 0.9). All results are detailed in Table 2.

**Table 2:** COV19‐QoL questionnaire results.

	Response^a^					
	1, % (*n*)	2, % (*n*)	3, % (*n*)	4, % (*n*)	5, % (*n*)	Mean ± SD
I think my quality of life is lower than before (*n* = 75)	0.0% (0)	9.3% (7)	18.7% (14)	37.3% (28)	34.7% (26)	4.0 ± 1.0
I think my mental health has deteriorated (*n* = 75)	2.7% (2)	18.7% (14)	21.3% (16)	44.0% (33)	13.3% (10)	3.5 ± 1.0
I think my physical health may deteriorate (*n* = 74)	1.4% (1)	4.1% (3)	14.9% (11)	55.4% (41)	24.3% (18)	4.0 ± 0.8
I feel more tense than before (*n* = 75)	0.0% (0)	10.7% (8)	21.3% (16)	50.7% (38)	17.3% (13)	3.7 ± 0.9
I feel more depressed than before (*n* = 74)	2.7% (2)	16.2% (12)	36.5% (27)	33.8% (25)	10.8% (8)	3.3 ± 1.0
I feel that my personal safety is at risk (*n* = 75)	14.7% (11)	22.7% (17)	29.3% (22)	26.7% (20)	6.7% (5)	2.9 ± 1.2

Abbreviation: COV19‐QoL, COVID‐19 Impact on Quality of Life.

^a^ 5‐point Likert scale: 1—Strongly disagree; 2—Disagree; 3—Neutral; 4—agree; and 5—strongly agree.

Of those who participated in the survey, 78.9% (*n* = 60) had outpatient appointments either cancelled/delayed or rescheduled during the pandemic across a range of clinical specialties and general practice. This is further detailed in Table 3.

**Table 3:** Participants' reports of the extent to which outpatient appointments were affected by the pandemic (survey 1).

	Participants who reported having appointments affected, % (percentage of survey respondents), *n* (number of people)
Appointments affected	78.9% (60)
Cancelled	30.0% (18)
Delayed	55% (33)
Rescheduled	61.2% (37)
Specialty appointment affected	
Rheumatology	58.8% (35)
Dentistry	31.7% (19)
General practice	31.7% (19)
Ophthalmology	21.7% (13)
Other^a^	36.7% (22)

^a^ Other: endocrinology, dermatology, haematology, orthopaedics, otolaryngology, oncology, radiology, nephrology, neurology.

Over the course of the pandemic, 17.1% (*n* = 12) of respondents had experienced disruptions to their prescribed medication regime, but none had reduced or discontinued their immunosuppression treatment over the course of the pandemic. A worsening of their sicca symptoms due to mask wearing was reported in 64.5% (*n* = 40) of survey respondents.

Further analysis indicated that there was a moderate correlation between total COV19‐QoL and ESSPRI scores (0.36, *p* = .002), and no statistically significant difference between the COV19‐QoL scores of those that had outpatient appointments affected during the pandemic and those that did not (*p* = .79).

### 3.4 Perspectives on telehealth

Fifty‐seven participants responded to the second survey focused on telehealth (response rate 29.4%). Of those, 70.2% (*n* = 40) of respondents had attended at least one telehealth appointment during the pandemic. These related to general practice (*n* = 28), rheumatology (*n* = 15), ophthalmology (*n* = 1) and others (*n* = 15). Overall, respondents reported positive experiences with telehealth (3.2 ± 1.1), with the majority indicating that telehealth was useful as it saved them time travelling to in‐person appointments (3.8 ± 0.9) and that they would use such services again (3.6 ± 0.9). However, most respondents felt that telehealth was not the same as in‐person visits (2.1 ± 1.0). The responses to the TUQ are detailed in Table 4 below.

**Table 4:** Modified TUQ questionnaire results.

	Response^a^					
	1, % (*n*)	2, % (*n*)	3, % (*n*)	4, % (*n*)	5, % (*n*)	Mean ± SD
Telehealth saves me time traveling to a hospital or specialist clinic (*n* = 39)	2.6% (*n* = 1)	5.1% (*n* = 2)	17.9% (*n* = 7)	20.5% (*n* = 8)	53.8% (*n* = 21)	3.8 ± 0.9
Telehealth improves my access to healthcare services (*n* = 38)	7.9% (*n* = 3)	10.5% (*n* = 4)	23.7% (*n* = 9)	5.3% (*n* = 2)	52.6% (*n* = 20)	3.4 ± 1.0
Telehealth provides for my healthcare needs (*n* = 39)	10.3% (*n* = 4)	20.5% (*n* = 8)	25.6% (*n* = 10)	2.6% (*n* = 1)	41.1% (*n* = 16)	3.1 ± 1.1
I could easily talk to the clinician using the telehealth system (*n* = 39)	2.6% (*n* = 1)	30.8% (*n* = 12)	10.3% (*n* = 4)	10.3% (*n* = 4)	46.2% (*n* = 18)	3.3 ± 1.1
I felt I was able to express myself effectively (*n* = 39)	5.1% (*n* = 2)	20.5% (*n* = 8)	12.8% (*n* = 5)	10.3% (*n* = 4)	51.3% (*n* = 20)	3.4 ± 1.1
I think the visits provided over the telehealth system are the same as in‐person visits (*n* = 39)	20.5% (*n* = 8)	56.4% (*n* = 22)	15.4% (*n* = 6)	0.0% (*n* = 0)	7.7% (*n* = 3)	2.1 ± 0.8
I feel comfortable communicating with the clinician using the telehealth system (*n* = 39)	2.6% (*n* =1)	25.6% (*n* = 10)	17.9% (*n* = 7)	0.0% (*n* = 0)	53.8% (*n* = 21)	3.2 ± 0.9
Telehealth is an acceptable way to receive healthcare services (*n* = 38)	5.3% (*n* = 2)	31.6% (*n* = 12)	21.1% (*n* = 8)	2.6% (*n* = 1)	39.5% (*n* = 15)	3.0 ± 1.0
I would use telehealth services again (*n* = 39)	5.1% (*n* = 2)	7.7% (*n* = 3)	17.9% (*n* = 7)	7.7% (*n* = 3)	61.5% (*n* = 24)	3.6 ± 0.9
Overall, I am satisfied with this telehealth system (*n* = 39)	7.7% (*n* = 3)	25.6% (*n* = 10)	12.8% (*n* = 5)	7.7% (*n* = 3)	46.2% (*n* = 18)	3.2 ± 1.2
Total	7.0% (*n* = 27)	23.5% (*n* = 91)	17.5% (*n* = 68)	6.7% (*n* = 26)	45.4% (*n* = 176)	3.2 ± 1.1

Abbreviation: TUQ, Telehealth Usability Questionnaire.

^a^ 5‐point Likert scale: 1—Strongly disagree; 2—Disagree; 3—Neutral; 4—Agree; and 5—Strongly agree.

**Figure 6:**
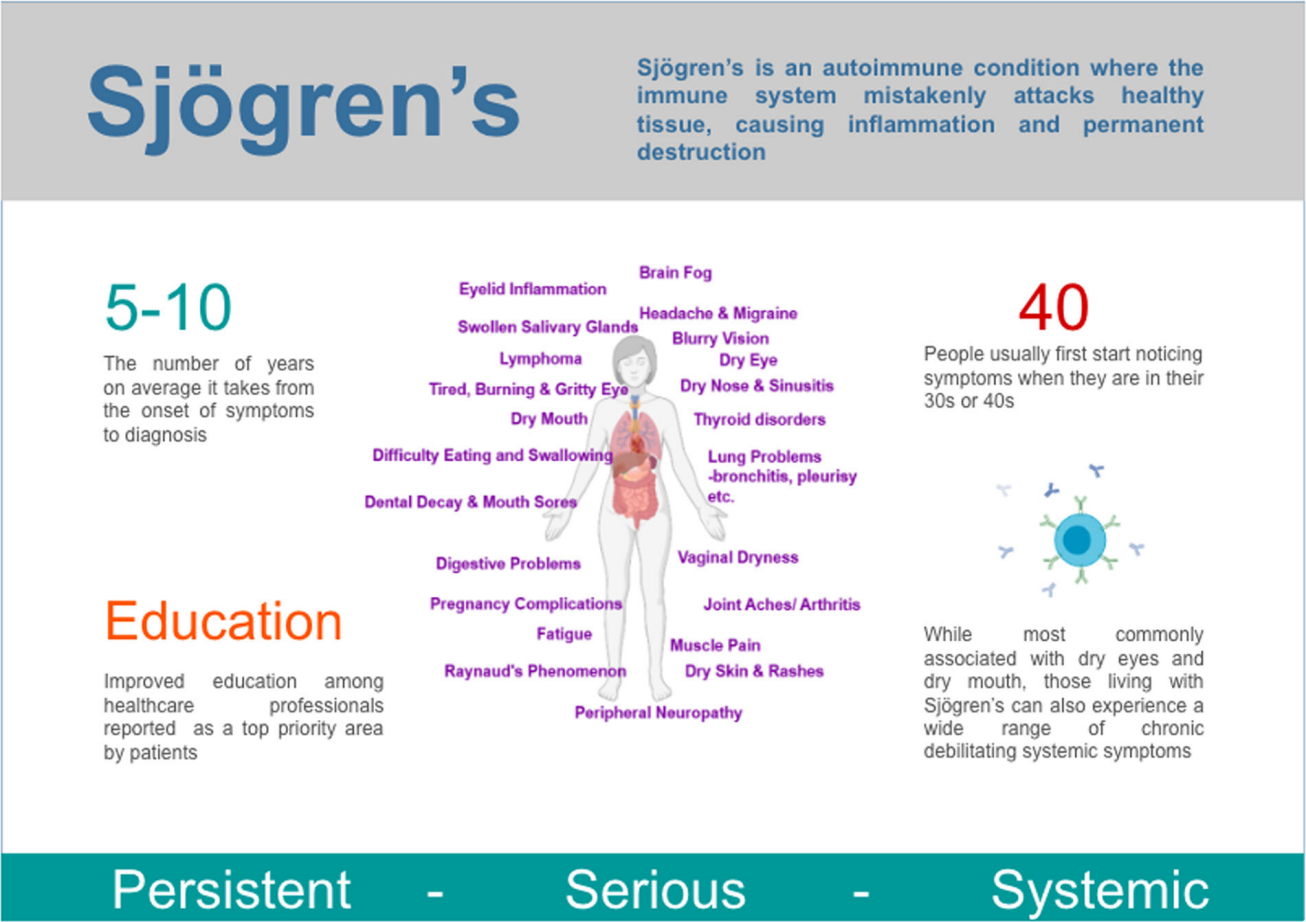
Summary of key study findings.

## 4 DISCUSSION

This study evaluates the reported experiences of patients living with Sjögren's in relation to symptoms, diagnosis and priorities, their experiences of the disease and healthcare during the COVID‐19 pandemic and perceptions of telemedicine.

Similar to other authors, we observed that the majority of participants experienced a significant delay between the onset of their symptoms and the diagnosis of their condition,^34^, ^35^, ^36^ with 41.4% of respondents waiting ≥5 years. Establishing a diagnosis is often challenging because of the condition's insidious and often heterogeneous presentation due to extra glandular involvement and/or comorbid autoimmune conditions, as well as a lack of physician awareness and accurate diagnostic tests (Figure 6). For those affected by Sjögren's, receiving a prompt diagnosis is essential for the instigation of appropriate treatment and optimal management of the disease. This is especially true in the case of those with pSS who are at greater risk of developing non‐Hodgkin's lymphoma as well as other cancers.^37^, ^38^, ^39^ A delay in diagnosis and the initiation of regular follow up for this subgroup can lead to a delay in the detection of malignancy.

Sjögren's can affect any part of the body. However, perhaps linked with the classification criteria, it is heavily associated with dry eyes and dry mouth with very little focus on the significant systemic component of the disease. To explore this further, we asked participants what disease symptoms they experienced daily. This generated a list containing over 100 unique responses which featured dry eye, dry mouth, fatigue, joint pain, dry skin, brain fog, muscle pain, vaginal dryness, neuropathy and headaches amongst the top symptoms experienced by participants daily. A recent study conducted by the Sjögren's Foundation in the United States to gain insights into the variety and severity of symptoms experienced by those living with Sjögren's determined that fatigue (79%), dry eyes (75%), dry mouth (73%), joint pain (65%), trouble sleeping (64%), eye discomfort (60%), muscle pain (56%) and brain fog (54%) had a major or moderate impact on their life.^40^ Interestingly, a comparison of the percentage of respondents reporting dry eye (95% vs. 74.4%), dry mouth (93% vs. 76.8%) and fatigue (88% vs. 57.3%) found that these symptoms were reported with higher frequency amongst the US survey participants compared to our study cohort. The differing timelines suggested (symptoms experienced during the past 12 months vs. daily) and means of data collection (a list of 48 symptoms provided via a free text box option) may offer some potential explanation for the apparent discrepancy in symptom frequency amongst these study cohorts. It is noticeable that to improve their medical care, participants of our study indicated that physician education was a top priority area. Our findings are supported by research conducted by Akpek et al., which determined that Sjögren's is underdiagnosed in patients with dry eye disease,^41^ suggesting a lack of awareness of disease symptoms amongst healthcare professionals. Furthermore, our findings are consistent with research conducted by Ngo et al. which found that there was a need to improve healthcare professional awareness of the seriousness of Sjögren's disease symptoms and the impact these symptoms have on patients' QoL.^42^

Due to the severe virulence of COVID‐19, containment was implemented through measures such as social distancing, lockdowns, cancelling of social gatherings, mask wearing, travel restrictions and work‐from‐home policies. While this physically reduced transmission of the virus, there is some evidence to suggest it placed a level of psychological stress and uncertainty upon the population that negatively impacts the wellbeing and QoL of some people.^43^, ^44^, ^45^ Furthermore, those with chronic diseases, including SS, are more susceptible to psychosocial stressors, both physically and psychologically.^5^, ^46^, ^47^ Respondents in this study indicated that the pandemic had worsened their symptoms of sicca, mirroring the findings of Carubbi et al. and also that it had negatively influenced their overall QoL and physical health and had left them feeling tenser than before.^48^ Additionally, those with a higher disease burden, as indicated through ESSPRI scores, perceived the pandemic as having a more negative impact on their QoL, as evidenced by their COV19 QoL scores. Similar findings have been reported by authors amongst patients with rheumatic diseases.^1^, ^2^

The worldwide pandemic has affected the delivery of routine care to patients with chronic illnesses due to government‐imposed restrictions and a diversion of resources to more acute COVID‐related care. This was reflected in our findings, where 79% of participants had medical appointments cancelled, delayed or rescheduled. Despite this disruption, however, there was no difference in ESSPRI or COV19 QoL scores between those who had appointments affected and those that did not, overall suggesting that Sjögren's patients had been able to adapt well to remote treatment options. Given the small sample size, additional studies are required to define if participants who had appointments cancelled/delayed, and so forth, had used telehealth.

COVID‐19 has further affected the delivery of healthcare through the increased use of telephone and online medical consultations in an effort to reduce transmission.^48^ This has drastically transformed the model of healthcare delivery, with the implementation of telehealth at both a speed and scale that could not have been conceived of before the pandemic. This impacted all aspects of care delivery including patient safety.^49^ More than 70% of respondents in this study had attended a telehealth consultation, reflecting this rapid and extensive change in the provision of care. Overall, those respondents had a positive experience with telehealth, and in particular indicated that virtual appointments saved them time, improved their access to healthcare and represented a service they would use again. As the pandemic continues with the continuing possibility of further restrictions and curtailment of routine care, it is apparent that we are going to have to learn to live with COVID‐19 and to develop a flexible method to continue to deliver healthcare and reduce risk to our most vulnerable patients. Thus, it is vital that patient and clinician satisfaction with telehealth is reviewed, audited and improved to ensure it can be integrated into our health service so as to have a positive impact on clinical outcomes and to assure the continued delivery of essential healthcare.

Utilising PPI through the involvement of patient advocates was integral to the development of the surveys used in this study and improving its appropriateness, design and reporting.^50^ We partnered with patient advocates, individuals who are ‘experts by experience’ in living with Sjögren's. Their unique experiential perspective was invaluable as part of the research team in seeking to design research questions and study outputs that would be genuinely inclusive, relevant and meaningful to people living with Sjögren's who participated in the survey.

## 5 LIMITATIONS

The results from our investigations should be viewed in light of the limitations of this study. One such limitation is our small sample size. The ongoing global pandemic at the time of participant recruitment is a potential factor leading to a lack of study respondents. Additionally, we have identified the delay from attendance at the webinar to the distribution of our surveys as another factor influencing study participation. However, by using a combination of widely validated questionnaires and bespoke items, we believe that this study offers a unique and important reflection of patient experiences, the impact of COVID‐19, and perceptions of telehealth of those living with Sjögren's.

Additionally, amongst the study's limitations, we acknowledge that this cross‐sectional study would not capture the ongoing effects of the COVID‐19 pandemic on all dimensions of QoL or attitudes towards telemedicine, nor can it predict long‐term repercussions on the health and wellbeing of those with Sjögren's. Furthermore, there is the potential for bias in our second towards participants who had experience with using telemedicine who may have strong negative or positive perceptions regarding its utility. The vast majority of respondents identified as female, and notwithstanding the fact that women are more likely to experience Sjögren's, it is possible that the findings may reflect a gender bias. While the purposive sampling approach employed ensured participants with the relevant experience were invited to take part, it may not have captured all perspectives, particularly those with limited access to technology.

## 6 CONCLUSION

We have captured the impact of Sjögren's on patient‐reported QoL, highlighting the delay between the presentation of symptoms and disease diagnosis, as well as the wide range of symptoms experienced by those affected beyond the pathognomonic triad of ‘dry eyes, dry mouth and fatigue’. Thus, we suggest, based on the lived experience of patients that for those involved in the treatment and management of this disease, Sjögren's should now more accurately be considered as a systemic multiorgan autoimmune disease associated with a chronic or progressive disease course characterised by secretory gland dysfunction in addition to a wide range of other systemic inflammatory manifestations (Figure 6). Furthermore, we have uniquely reported the effect of the COVID‐19 outbreak on the delivery of routine care to patients with Sjögren's, and their overall positive attitude to telemedicine alternatives to face‐to‐face consultations. There is a need to enhance the flexibility and responsiveness of healthcare when faced with COVID‐19 and other infections, individual patient needs and possible future pandemics.

## AUTHOR CONTRIBUTIONS

The authors confirm their contribution to the paper as follows: *Study conception and design*: Joan Ní Gabhann‐Dromgoole, Emily Greenan and Deirdre Collins. *Data collection*: Joan Ní Gabhann‐Dromgoole. *Analysis and interpretation of results*: Joan Ní Gabhann‐Dromgoole, Emily Greenan, Deirdre Collins, Gráinne Tynan, Conor C. Murphy and Michelle Flood. *Draft manuscript preparation*: Joan Ní Gabhann‐Dromgoole, Emily Greenan, Gráinne Tynan and Michelle Flood. All authors reviewed the results and approved the final version of the manuscript.

## CONFLICT OF INTEREST STATEMENT

The authors declare no conflict of interest.

## Data Availability

The data that support the findings of this study are available from the corresponding author upon reasonable request.
